# Zeroing in on xylazine - a mixed methods study to explore correlates of reported xylazine use and educational gaps among people who use drugs

**DOI:** 10.1186/s12954-026-01437-7

**Published:** 2026-04-03

**Authors:** Katherine Hill, Rebecca Minahan-Rowley, Emma Biegacki, Robert Heimer, Kimberly L. Sue

**Affiliations:** 1 Department of Epidemiology of Microbial Diseases, Yale School of Public Health, New Haven, USA; 2 Yale Program in Addiction Medicine, Yale School of Medicine, New Haven, USA; 3 Department of General Internal Medicine, Yale School of Medicine, New Haven, USA

**Keywords:** Harm reduction, Xylazine, Fentanyl, Substance use, Opioids, Education

## Abstract

**Background:**

Xylazine prevalence has surged in the unregulated fentanyl supply across the United States. In efforts to inform education initiatives for people who use drugs, this study aimed to identify factors associated with reported xylazine use and knowledge gaps.

**Methods:**

This Connecticut-based mixed methods study employed both surveys and interviews. Survey participants, recruited from collaborating harm reduction organizations, were adults who used unregulated substances in the last six months. They were asked about their drug use, knowledge of xylazine, service use experience, and demographic information. Purposive sampling of survey participants was then used to select individuals for in-depth interviews. Topic guides were designed to gain a deeper understanding of participants’ xylazine awareness and future learning preferences. Quantitative data were dichotomized based on reported xylazine use and analyzed using descriptive statistics, Chi-square, Fisher’s exact, or t-tests. Simple and multiple logistic regression were used to find correlates of reported use of xylazine. Interviews were analyzed using thematic analysis.

**Results:**

Between August and November 2024, we surveyed 114 respondents. Nearly half reported lifetime xylazine use (49.1%) and almost three-quarters heard of xylazine prior to engaging in the study (71.9%). Multiple logistic regression revealed that unemployment (OR 6.5, 95% CI 2.4–17.8), past-year fentanyl use (OR 41.1, 95% CI 4.8–354.4), and harm reduction service engagement (OR 5.2, 95% CI 1.6–16.8) as predictors of reported xylazine use, after adjustments. We conducted n=31 interviews; thematic analysis identified four major themes: (1) information about xylazine was obtained from many sources, (2) knowledge about xylazine was gained from using xylazine, (3) some misconceptions about xylazine have developed, and (4) multiple mechanisms for xylazine education are desired.

**Conclusions:**

This study underscores the need for accessible xylazine education to address knowledge and confidence gaps among people who use drugs. Xylazine education should be tailored to one’s likely xylazine exposure. Education must be harm-reduction oriented, stigma-free, and incorporate the voices of people who use drugs.

**Supplementary Information:**

The online version contains supplementary material available at 10.1186/s12954-026-01437-7.

## Background

Xylazine, an alpha-2 adrenergic receptor agonist, has become increasingly prevalent in the unregulated fentanyl supply across the United States [1–4]. This substance, never approved for use in humans by the Food and Drug Administration, is a veterinary sedative commonly used surgically [4–6]. Thus, the rise of xylazine in the unregulated market has raised numerous concerns within the harm reduction and addiction medicine fields, and introduced challenges for people who use drugs (PWUD) related to overdose, intense sedation, slow-healing wounds, and withdrawal [1, 7–10]. Collectively, these constellation of effects have been called xylazine-related outcomes (XROs) [11].

In Connecticut (CT), xylazine first appeared in overdose toxicology reports in 2019, and has steadily appeared in approximately 25% of fentanyl-involved overdose deaths from 2021-2023 [12, 13]. Beyond overdose, other XROs have also impacted PWUD in our state; for instance, necrotic wounds among those who have been exposed to xylazine in their drugs have also been observed in patients [14, 15]. Given the rise in xylazine prevalence, efforts across the state have sought to increase access to services that can help PWUD manage xylazine exposure (e.g., drug checking, wound care). Previous studies by our research team have demonstrated awareness and knowledge about xylazine and XROs among both harm reductionists and harm reduction-oriented healthcare providers in CT [11, 16]. However, gaps still exist regarding the information that medical care providers and harm reductionists need to share with people exposed to xylazine, as well as the appropriate timing and locations for disseminating this information [11, 16].

This study aimed to explore the factors associated with xylazine use/exposure and expand knowledge and confidence gaps among CT-based PWUD to better target educational efforts around xylazine and XROs.

## Methods

### Study design and aims

This mixed methods study was conducted in collaboration with harm reduction organizations around CT using a quantitative survey and follow-up qualitative one-on-one interviews. Such a design allowed us not only to rapidly gain a deeper understanding of PWUD’s knowledge of xylazine and confidence in managing exposures to xylazine but also to home in on potential interventions by which PWUD can come to learn more about xylazine and XROs.

### Participants and setting

We aimed to recruit 95 survey participants for this study through convenience sampling via collaboration with seven CT organizations that serve PWUD. This number was established to ensure sufficient geographic and demographic variety across the participating programs. Participants, recruited on-site at participating organizations, were eligible if they had used unregulated substances within the last six months, were aged 18 or older, and lived in CT. We allotted the number of survey slots made available at each location based on *a priori* decisions about where xylazine prevalence was known to be highest (e.g., our Hartford location was given more survey slots than the one in Middletown). Among those who completed a survey and were interested in a subsequent interview, we utilized purposive sampling via following axes of selection: (1) site location; and (2) self-reported xylazine exposure. We planned to conduct in-depth interviews with a select set of participants until theoretical saturation was reached, which we estimated *a priori* from previous research studies to be approximately n=30.

### Study instruments

Our short survey, designed to take approximately 10 minutes to complete, consisted of 36 closed-ended questions covering topics including substance use history and preferences, use and knowledge of xylazine, confidence around managing xylazine exposure, service use experience, demographic information, and interest in an interview. The questions on substance use history and preferences and participant demographics have been used by our research team in previous surveys [16]. We adapted the xylazine-specific questions based on our recent study on healthcare provider xylazine knowledge [11].

Our semi-structured interview guide for in-depth interviews contained 25 questions, each with a series of follow-up questions or probes based on participant responses and was designed to take approximately 45 minutes to complete. The guide covered the following topics: healthcare and harm reduction service use, awareness of xylazine, impact of xylazine on drug use, XROs (i.e., wounds, sedation, withdrawal, overdose), and xylazine education preferences.

### Data collection procedures

When clients of collaborating organizations entered the participating site for services (e.g., getting syringes, a methadone dose, etc.), organizational and/or research staff would ask if they might also be interested in participating in a voluntary, remunerated research study during their visit. Those who expressed interest in participating were taken into a private space (e.g., an office, an exam room, etc.) to help ensure participant confidentiality. Potential participants were then screened for eligibility and provided study information including the purpose of the study, what information would be collected, and how long the study might take them. Participants had the option to respond verbally to the survey read aloud to them or to complete the survey on their own via a tablet or on paper. Participants choosing the former provided consent verbally. Participants choosing the latter provided consent by selecting “Yes” to the consent question. Participation was voluntary and individuals could withdraw from the study at any time without penalty. Ultimately, we collected n=114 surveys utilizing a web-based software platform, Research Electronic Data Capture (REDCap). Participants were provided with $10 cash for their participation in the survey portion of the study.

Among those who were interested in a follow-up interview (n=72, 63.7%) and consented to audio-recording (n=69, 95% of those who were interested), we selected participants via purposive sampling; we planned to interview participants proportionately to the number of surveys conducted in a given location, with a ratio of 1 interview for every 3–4 surveys conducted at a given study site. We also selected participants on the axis of reported xylazine exposure; for instance, if someone was intentionally or accidentally using xylazine daily they were more likely to be invited for an interview than someone who reported never using xylazine. We interviewed n=31 participants, at which point our research team agreed we reached theoretical saturation. All interviewees were compensated an additional $30 cash, for a total of $40 from participation in the survey and interview portions.

### Data analysis procedures

All quantitative survey data were summarized using descriptive statistics for the full survey population and stratified by reported exposure to xylazine. Reported exposure to xylazine was the primary outcome; this variable was dichotomized based on responses to the question “Have you ever used xylazine or drugs that contain xylazine?” Those who selected “Yes—I have used xylazine intentionally” or “Yes—I have used xylazine but by accident” were collapsed into the “Yes” group and those who selected “No” or “I am not sure” were collapsed into the “No” group. To compare differences between these two groups for selected characteristics, Chi-square or Fisher’s exact tests were used- based on cell sizes—for categorical variables while t-tests were used for continuous variables. We utilized simple and multiple logistic regression to find correlates of reported exposure to xylazine; variables were selected manually for inclusion in multiple regression based on statistical significance in bivariate testing and backward selection was used iteratively until only statistically significant predictors remained (pre-determined to be α=0.05). All quantitative analyses were performed using SAS Software, Version 9.4.

Interviews were audio-recorded using the teleconferencing software platform, Zoom. Raw transcripts from this platform were then cleaned and verified for accuracy by members of the research team. All identifying information was removed from transcripts, filler words were removed as appropriate (e.g. “uhm”), and indications of body movement and expression were noted where possible (e.g. *[laughing]*). All transcripts were coded using NVivo software, version 12 (QSR International) for thematic analysis [17]. This was based on a codebook created and iteratively improved by the research team; themes were then brainstormed, developed, and discussed. Pseudonyms for respondents were created for all qualitative analyses presented below.

Lastly, the quantitative and qualitative data analyses were then integrated to explore emerging trends in the data and identify patterns between the datasets. We utilized case-by-case analysis where appropriate to understand why participants might have certain knowledge gaps and parallel comparisons to build connections between ‘what’ participants are experiencing (i.e., survey results) and ‘why’ they might be experiencing these things (i.e., qualitative results).

### Funding and human subjects protections

This study was funded by the Yale New Haven Hospital Medical Staff Fund. All materials and study procedures were approved by Yale Human Subjects Protections Program (Protocol #2000036264).

## Results

### Survey results

Between August 28th and November 7th, 2024, we collected n=114 unique survey responses—n=19 more than originally planned, due to the interest and availability of study participants. Almost half of our total sample reported using xylazine in their lifetime (49.12%, n=56).

#### Participant socio-demographic characteristics

All socio-demographic information for the full sample and stratified by the primary outcome (i.e., reported xylazine use) can be found in Table 1. Study participants were 48.7 years old on average (SD=11.9 years), with those with reported xylazine use slightly younger on average than those without xylazine use (45.3 years and 51.9 years, respectively; *p*=0.003). Regarding gender identity, 69.3% of the sample identified as male, with no statistically significant differences between those who had and hadn’t used xylazine. The sample was racially and ethnically diverse, with 54.4% identifying as White (n=62), 28.1% identifying as Black or African American (n=32), and 25.7% identifying as Hispanic or Latino (n=29); there were no statistically significant differences between those who had and hadn’t used xylazine for race and ethnicity. Most participants had low educational attainment with a high school degree or less (n=76, 66.7%), were unemployed, disabled, or retired (n=97, 85.1%), and were homeless or temporarily living with a family member or friend (n=66, 57.9%). Of note, there were statistically significant differences between employment status for the primary outcome; prevalence of unemployment was much higher among those with reported xylazine use than those who did not (73.2% vs. 53.4%, *p*<0.001).

**Table 1:** Demographic characteristics among those using unregulated substances in last 6 months, stratified by reported use of or exposure to xylazine

	Full Sample (N=114)		Reported use of xylazine (n=56)		No reported use of xylazine (n=58)		
Characteristics	Frequency	%	Frequency	%	Frequency	%	*p*-value
Age (n=112)							**0.003**
Mean (SD)	48.72	11.94	45.28	11.51	51.93	11.52	
Gender							1.000
Male	79	69.30	39	69.64	40	68.97	
Female	34	29.92	17	30.36	17	29.31	
Other gender	1	0.88	0	0.00	1	1.72	
Race*							
White	62	54.39	33	58.93	29	50.00	0.339
Black or African American	32	28.07	13	23.21	19	32.76	0.257
Native American / Alaskan Native	4	3.51	1	1.79	3	5.17	0.619
Other race	22	19.30	10	17.86	12	20.69	0.702
Ethnicity							0.961
Hispanic or Latino	29	25.66	14	25.45	15	25.86	
Not Hispanic or Latino	84	74.34	41	74.55	43	74.14	
Recruitment location							**<0.001**
Bridgeport	27	23.68	11	19.64	16	27.59	
Hartford	25	21.93	12	21.43	13	22.41	
New Haven	20	17.54	16	28.57	4	6.90	
New London	20	17.54	11	19.64	9	15.52	
Middletown	15	13.16	1	1.79	14	24.14	
Torrington	7	6.14	5	8.93	2	3.45	
Highest level of education (n=112)							0.699
Less than a high school degree	34	30.36	15	27.78	19	32.76	
High school degree or equivalent	42	37.50	19	35.19	23	39.66	
Associate degree	8	7.14	6	11.11	2	3.45	
Some college but no degree	23	20.54	12	22.22	11	18.97	
Bachelor degree	3	2.68	1	1.85	2	3.45	
Graduate degree	2	1.79	1	1.85	1	1.72	
Employment status							**<0.001**
Unemployed (Looking for work)	40	35.09	28	50.00	12	20.69	
Unemployed (Not looking for work)	20	17.54	13	23.21	7	12.07	
Disabled	32	28.07	8	14.29	24	41.38	
Employed (1-39 hours per week)	9	7.89	4	7.14	5	8.62	
Employed (40+ hours per week)	8	7.02	2	3.57	6	10.34	
Retired	5	4.39	1	1.79	4	6.90	
Living arrangement (n=113)							0.216
Lives alone	54	47.79	23	41.82	31	53.45	
Lives with other people	59	52.21	32	58.18	27	46.55	
Living situation							1.000
In a home	47	41.23	23	41.07	24	41.38	
In a residential facility	1	0.88	1	1.79	0	0.00	
Staying in a shelter / homeless	55	48.25	27	48.21	28	48.28	
Staying with a relative or friend	11	9.65	5	8.93	6	10.34	

^*^Will not sum to 100%, as participants were able to select multiple choices

Bolded values are statistically significant

#### Participant recruitment location

Based on our *a priori* recruitment locations decisions, we recruited the most participants in Bridgeport (n=27) and the fewest participants in Torrington, CT (n=7). Table 1 demonstrates that the primary outcome differed by recruitment location (*p*<0.001), with those in New Haven and Middletown having the highest and lowest prevalence of xylazine use, respectively. Supplemental Figure 1 provides an overview of where participants lived, based on self-reported zip codes, alongside a heat map of xylazine-involved fentanyl overdose deaths from 2019 to 2023 (provided by the CT Office of the Chief Medical Examiner).

#### Participant substance use history

Table 2 provides a summary of participants’ substance use history, tabulated for the full sample and stratified by the primary outcome. Our sample was largely composed of those with many years of experience with substance use, as the average number of years using substances in our sample was 23.4 (SD=12.9). Additionally, there was a diversity of substances used in the last year among the study sample, with crack cocaine (n=91, 79.8%) and fentanyl (n=88, 77.2%) being the most commonly used substances. There were differences in the prevalence of endorsing specific drugs used in the last year between those reporting xylazine use and those without; fentanyl (*p*<0.001), heroin (*p*=0.002), benzodiazepines (*p*<0.001), and other drugs such as LSD and PCP (*p*=0.032), were more commonly endorsed for past-year use among those with reported xylazine use than those without. For participants’ preferred drug in the last year, fentanyl was the most commonly endorsed substance (n=34, 30.1%). Further, when comparing the preferred drug based on reported xylazine use, there were statistically significant differences between groups (*p*<0.001); of note, those with reported xylazine use more commonly reported fentanyl as their preferred substance than those without xylazine use (47.3% and 13.8%, respectively). Lastly, for the full sample, smoking was the most common route of drug administration (n=101, 88.6%). However, injecting drugs was more common among those reporting than those not reporting xylazine use (66.1% and 31.0%, respectively; *p*<0.001).

**Table 2:** Substance use history and practices in the last year, stratified by reported use of or exposure to xylazine

	Full sample (N=114)		Reported use of xylazine (n=56)		No reported use of xylazine (n=58)		
Characteristics	Frequency	%	Frequency	%	Frequency	%	*p*-value
Number of years using substances							0.097
Mean (SD)	23.36	12.88	21.32	12.02	25.33	13.47	
Drug use in last year*							
Crack Cocaine	91	79.82	45	80.36	46	79.31	0.889
Fentanyl	88	77.19	55	98.21	33	56.90	**<0.001**
Cannabis	83	72.81	37	66.07	46	79.31	0.112
Heroin	79	69.30	48	85.71	31	53.45	**0.002**
Powder Cocaine	55	48.25	31	55.36	24	41.38	0.135
Benzodiazepines	49	42.98	33	58.93	16	27.59	**<0.001**
Pharmaceutical Medications	37	32.46	23	41.07	14	24.14	0.054
Methamphetamine	19	16.67	12	21.43	7	12.07	0.180
Other**	16	14.04	12	21.43	4	6.90	**0.032**
Drug use method in last year*							
Orally	66	57.89	36	64.29	30	51.72	0.175
Smoking	101	88.60	50	89.29	51	87.93	0.820
Snorting	72	63.16	37	66.07	35	60.34	0.526
Injecting	55	48.25	37	66.07	18	31.03	**<0.001**
Boofing	2	1.75	2	3.57	0	0.00	0.239
Preferred drug in last year (n=113)							**<0.001**
Fentanyl	34	30.09	26	47.27	8	13.79	
Crack Cocaine	30	26.55	9	16.36	21	36.21	
Heroin	28	24.78	15	27.27	13	22.41	
Cannabis	10	8.85	1	1.82	9	15.52	
Powder Cocaine	5	4.42	1	1.82	4	6.90	
Other**	2	1.77	0	0.00	2	3.45	
Pharmaceutical Medications	2	1.77	2	3.64	0	0.00	
Methamphetamine	1	0.88	0	0.00	1	1.72	
Benzodiazepines	1	0.88	1	1.82	0	0.00	
Engaged in harm reduction services in last year							**<0.001**
Yes	81	71.05	48	85.71	33	56.90	
No	33	28.95	8	14.29	25	43.10	
Engaged in healthcare in last year							0.436
Yes	89	78.07	42	75.00	47	81.03	
No	25	21.93	14	25.00	11	18.97	
Talked to provider about drug use in last year (n=89)							0.096
Yes	67	75.28	35	83.33	32	68.09	
No	22	24.72	7	16.67	15	31.91	

^*^ Will not sum to 100%, as participants were able to select multiple choices

^**^ Other drugs noted by participants included: LSD, MDMA, Angel Dust, Mushrooms, Molly, Ecstasy, Acid, K2, PCP, Tusi

Bolded values are statistically significant

#### Participant service use history

Additionally, Table 2 provides an overview of service use among the study sample. Most participants had seen a healthcare provider in the last year (n=89, 78.1%), and among those, most participants had talked to this provider about their drug use (n=67, 75.3%). Healthcare service use did not differ based on reported xylazine use. Regarding past-year engagement in harm reduction services, a smaller but still large proportion of our study sample accessed and utilized these services (n=81, 71.1%). However, those with xylazine use had a higher prevalence of service engagement than those not reporting xylazine use (85.7% and 56.9%, respectively; *p*<0.001).

#### Participant knowledge about xylazine

Table 3 tabulates participants’ awareness and knowledge about xylazine in the unregulated drug supply and XROs. Among our survey sample, 71.9% of participants (n=82) had heard of xylazine prior to engaging in the research study; those with reported use of xylazine endorsed that they had heard about xylazine more often than those without reported use of xylazine (*p*<0.001). Regarding each statement participants had to evaluate, we determined how many people answered each question correctly compared to those answering incorrectly or who selected “I do not know.” Overall, less than two-thirds of the sample knew it was possible to test drugs for xylazine (n=71, 62.3%) and that xylazine is associated with severe wounds that are difficult to heal (n=69, 60.5%). Just over half of the sample knew that someone who uses xylazine frequently may experience withdrawal from it (n=67, 58.8%), that xylazine is typically added to opioids to make the high seem longer (n=63, 55.3%), and that xylazine is more often found in fentanyl than other substances (n=63, 55.3%). Small proportions of the study sample knew that naloxone cannot reverse sedative effects of xylazine (n=41, 36.3%) and that xylazine decreases heart rate (n=27, 23.7%). Ultimately, for all seven knowledge-based questions asked of participants, those with reported use of xylazine had a higher percentage of correct answers than those with no reported use of xylazine; further, six of these questions had a statistically significant difference between the primary outcome groups.

**Table 3:** Xylazine-related knowledge and confidence among those with past year unregulated substance use, stratified by reported use of or exposure to xylazine

	Full sample (N=114)		Reported use of xylazine (n=56)		No reported use of xylazine (n=58)		
Characteristics	Frequency	%	Frequency	%	Frequency	%	*p*-value
*Previously heard of xylazine*							
Yes	82	71.93	53	94.64	29	50.00	**<0.001**
*Xylazine related knowledge*							
Knew it is possible to test drugs for xylazine	71	62.28	43	76.79	28	48.28	**0.002**
Knew xylazine is associated with severe wounds that are difficult to heal	69	60.53	45	80.36	24	41.38	**<0.001**
Knew that someone who uses xylazine frequently may experience withdrawal from it	67	58.77	38	67.86	29	50.00	0.053
Knew xylazine is typically added to make the opioid high seem longer	63	55.26	42	75.00	21	36.21	**<0.001**
Knew xylazine is more often found in fentanyl than other substances	63	55.26	45	80.36	18	31.03	**<0.001**
Knew naloxone cannot reverse sedative effects of xylazine	41	36.28	31	56.36	10	17.24	**<0.001**
Knew xylazine decreases heart rate	27	23.68	23	41.07	4	6.90	**<0.001**
*Confidence around managing xylazine and xylazine related outcomes*							
Had confidence in knowing whether xylazine was in their drugs	61	53.51	38	67.86	23	39.66	**0.003**
Had confidence in recognizing xylazine's side effects	73	64.04	46	82.14	27	46.55	**<0.001**
Had confidence in knowing where to seek treatment for xylazine's side effects	78	68.42	38	67.86	40	68.97	0.899
Had confidence in ability to prevent potential bad side effects related to xylazine	60	53.09	30	53.57	30	52.63	0.920

Bolded values are statistically significant

#### Participant confidence in managing xylazine in the unregulated supply

Additionally, Table 3 provides a summary of whether participants felt confident in managing aspects of having xylazine in the drug supply, wherein ‘confidence’ encompassed those feeling slightly, somewhat, quite, or extremely confident. There was relatively low confidence for all survey items addressed. About half of the participants had confidence in knowing whether xylazine was in their drugs (n=61, 53.5%); with a higher prevalence of confidence for this item among those with reported use of xylazine compared to those without reported use (67.9 and 39.7%, respectively; *p*=0.003). A higher proportion of participants had confidence in recognizing xylazine’s side effects (n=73, 64.0%) and in knowing where to seek treatment for such side effects (n=78, 68.4%). Those with reported xylazine use were more likely to be confident in recognizing xylazine's side effects than those without reported xylazine use (82.1% and 39.7%, respectively; *p*<0.001). Consistently, regardless of the primary outcome, approximately 53% of the participants had confidence in their ability to prevent potential bad side effects related to xylazine (n=60).

#### Logistic regression

Simple and multiple logistic regression models exploring correlates of the primary outcome can be found in Table 4. Our multiple regression model adjusted for employment status, past-year fentanyl use, and past-year engagement in harm reduction services. Those who were unemployed had 6.49 times the odds of reported xylazine use compared to those who were employed, disabled, or retired (95% CI: 2.37, 17.80), adjusting for other factors in the model. There was also a very robust correlation between past-year fentanyl use and reported xylazine use, wherein those who used fentanyl in the last year had 41.10 times the odds of reported xylazine use compared to those who did not use fentanyl in the last year (95% CI: 4.77, 354.42), adjusting for other factors in the model. Those engaged in harm reduction services had 5.24 times the odds of reporting xylazine use compared to those who were not engaged in harm reduction services (95% CI: 1.64, 16.77), adjusting for other factors in the model.

**Table 4:** Correlates of reported xylazine use

	Unadjusted OR		Adjusted OR	
Correlates	OR	95% CI	OR	95% CI
*Age*				
1-Year	0.95	(0.92, 0.98)		-
*Recruitment location*				
New Haven vs. Middletown	55.99	(5.58, 561.49)	-	-
Torrington vs. Middletown	34.99	(2.58, 475.10)		
New London vs. Middletown	17.12	(1.87, 156.18)	-	-
Hartford vs. Middletown	12.92	(1.47, 113.72)		
Bridgeport vs. Middletown	9.62	(1.10, 84.19)	-	-
*Employment status*				
Unemployed vs. Other	5.61	(2.51, 12.57)	6.72	**(2.43, 18.60)**
*Drugs used in last year*				
Fentanyl Use vs. None	41.67	(5.39, 321.99)	39.54	**(4.48, 341.36)**
Heroin Use vs. None	5.25	(2.11, 12.97)	-	-
Benzodiazepenes vs. None	3.77	(1.72, 8.25)	-	-
Other Drug Use vs. None	3.68	(1.11, 12.22)	-	-
*Drug use method in last year*				
Injecting vs. Not Injecting	4.33	(1.97, 9.48)		-
*Preferred drug in last year*				
Fentanyl preferred vs. other	5.42	(2.18, 13.49)		-
*Engaged in harm reduction services in last year*				
Yes vs. No	4.55	(1.83, 11.31)	5.02	(1.56, 16.13)

Bolded values are statistically significant

### Interview results

We conducted n=31 semi-structured interviews; the average interview lasted approximately 35 minutes. Table 5 contains a summary of the demographic characteristics of interview participants, who closely mirrored the survey participants. Purposive sampling by site location and self-reported xylazine exposure was successful; 5 of 6 study sites were represented in similar numbers (i.e., between 4 and 8 participants) and most participants (n=27) reported lifetime exposure to xylazine. Qualitative thematic analysis revealed four primary themes informing what PWUD might be seeking when it comes to xylazine education initiatives, and the knowledge and confidence gaps they might have related to xylazine and XROs. Pseudonyms for participants are used below.

**Table 5:** Demographic characteristics among interview participants

Characteristics	Interview participants	
	(n=31)	
	Frequency	%
*Age (n=30)*		
Mean (SD)	44.63	11.04
*Gender*		
Male	20	64.52
Female	11	35.48
Race*		
White	18	58.06
Black or African American	7	22.58
Other Race	7	22.58
*Ethnicity*		
Hispanic or Latino	24	77.42
Not Hispanic or Latino	7	22.58
*Recruitment location*		
Bridgeport	7	22.58
Hartford	8	25.81
New Haven	5	16.13
New London	7	22.58
Torrington	4	12.90
*Highest level of education (n=30)*		
Less than a high school degree	9	29.03
High school degree or equivalent	9	29.03
Associate degree	4	12.90
Some college but no degree	7	22.58
Graduate degree	1	3.23
*Employment status*		
Unemployed (Looking for work)	15	48.39
Unemployed (Not looking for work)	8	25.81
Disabled	5	16.13
Employed (1-39 hours per week)	2	6.45
*Living arrangement (n=30)*		
Lives alone	13	41.94
Lives with other people	17	54.84
*Living situation*		
In a home	13	41.94
Staying in a shelter / homeless	14	45.16
Staying with a relative or friend	4	12.90

^*^ Will not sum to 100%, as participants were able to select multiple choices

#### Theme 1: Participants learned about xylazine through various mechanisms

Participants expressed that they had heard of and learned about xylazine through a multitude of mechanisms, including (a) word of mouth, (b) during service provision, (c) in the media, and/or (d) on the internet. In this way, it is possible that education initiatives through various mechanisms may successfully reach PWUD as a target audience.

##### Subtheme 1.A. Word of mouth

Most commonly, participants noted that they heard about xylazine in their communities through word of mouth from others who use drugs. For instance, Barbara, a White woman in her sixties, had first heard of xylazine approximately three years ago when she saw a xylazine-associated wound on a young girl in her community. She explained,

*“When I first heard about [xylazine] it was because I noticed her arm. And then she explained to me what it was… I asked her a lot of questions... When I seen her, I was like, ‘what the hell is that?’ I've never seen that before… She’s the one that explained to me that they're putting xylazine in the heroin. And then she explained to me why her arms look the way they did, because I says, ‘I really just want to know, I don't want to make you feel uncomfortable, just totally curious, you know?’ Then I explained to her that when I was shooting dope, people used to get abscesses. Hers aren’t an abscess. It's a sore.”*

A few months after learning about xylazine and its association with wounds, Barbara noticed a burning sensation on her upper lip, which she strongly believed was from snorting fentanyl with xylazine in it—especially since she would “fall into a deep sleep” after using and “the [test] strips show positive for xylazine.” In this way, Barbara was equipped with knowledge that let her know (a) xylazine was in her drug supply, (b) what was happening to her body potentially as a result of xylazine, (c) how to check for xylazine in her drugs of choice, and (d) what to do if she got a wound; in fact, Barbara eventually went to the emergency room to ask for the same ointment the young girl in her neighborhood used previously.

##### Subtheme 1.B. Service provision

Other participants described that the first time they learned about xylazine was while receiving services, such as obtaining syringes in a harm reduction center, getting their methadone dose, or attending a class at rehab. This included (a) seeing pamphlets and flyers, either on waiting room tables or posted onto cork boards, (b) talking to program staff while getting supplies, or (c) while seeing a clinician. Certainly, seeing a clinician was not always a pleasant experience for participants who were in active use; some participants experienced fear, stigma, anger, mistrust, and the like from their providers—especially in the context of a novel drug that many participants’ providers knew little about and for which standards of care were evolving. However, other participants described having positive experiences engaging with clinicians who seemed more knowledgeable. For instance, some participants expressed they could learn from clinicians about xylazine and its impacts. Consider the case of Nolan, a White man in his thirties. After using fentanyl intravenously for about 5 years, he started to struggle with new skin wounds he thought might be linked to xylazine. He explained,

*“About a year ago, I'd gone to the hospital with one of those abscesses, and the doctor? He comes in and he started explaining to me about xylazine and the effects. And he's like ‘These lesions, they look just like abscesses, behave like abscesses, but they're not. It's not resulting from an infection. It's resulting from the xylazine.’ And he goes, ‘They’re a real bitch.’ That’s exactly how the doctor put it.”*

Not only did Nolan value learning about xylazine and its impacts from this provider, but he also expressed enjoying this provider’s straightforward and candid attitude about xylazine and its potential impact (i.e., wounds). In fact, many participants indicated that they wanted healthcare and harm reduction service providers to not ‘sugar coat’ what PWUD might face when it came to XROs. Participants expressed a deep desire to understand what xylazine was doing to their body.

##### Subtheme 1.C. Through the media

Fewer participants described that they learned about xylazine through television or social media. However, the experience of hearing about xylazine in the media was often not only informative, but also reflective in that participants had the opportunity to see themselves on the screen. For instance, Rodney, a Black man in his mid-fifties, had first heard about xylazine “on the news” about a year ago. He described his response to the television show, by stating,

*“When I heard about it? Every. Symptom. I. Had. I used to stand up, I'm sleeping and don't know it!”*

After over thirty years of experience using heroin, Rodney could not make sense of why he was suddenly slumped over while standing and extremely sedated after use. One night, images on the television screen answered this for him—xylazine. This moment was a turning point for Rodney, as he was able to search for the term online, ask questions at his local syringe service program, and talk to his community about this new drug. This story was not unique; other participants mentioned seeing videos of places like Kensington in Philadelphia, PA on social media platforms like YouTube or TikTok. Participants did not seem to be upset by the content of these videos; instead, they were more so comforted knowing that they were not alone and that they finally had answers to what they had been experiencing.

##### Subtheme 1.D. On the internet

Participants were not just passively learning about xylazine. Most, if not all, participants described how they were self-motivated to independently search and learn about xylazine online when they had the time and opportunity. Participants were incredibly curious about this new drug in the supply (e.g., Why does it cause these wounds? Can you overdose on it? What would withdrawal be like?). Participants described searching for information not just in the places previously mentioned, but also on the Internet: Google, Reddit, Quora, etc. For instance, Marvin, a Black man in his early sixties, explained,

*“I like looking up shit. I tried to look up to see what the fuck everyone is doing out here that got them fucked up like me. That is how I learned about it.”*

In this way, participants were able to search for stories from people with experiences that mirrored their own. This allowed them to find comfort in answers that they could not find in their own circles, expanding their education on xylazine beyond the knowledge contained by the people or the service locations around them.

#### Theme 2: Participants learned about xylazine from using xylazine

While many participants had the advantage of hearing about xylazine before using the drug, giving them time to prepare for it entering their local supply, other participants were, unfortunately, less prepared. Jamila, a woman in her early fifties who preferred to not disclose her race, has been using fentanyl and crack for approximately 5 years; she explained,

*“I was using [xylazine] long before I heard about it. I didn't know about it. I didn't know of it. It was just in my shit… It is this stuff that makes you nod out. It's just real strong shit… It's like being sleepy on steroids. Definitely. That’s the only way I can make you understand it. It's like being sleepy, but on steroids. The most sleepy you've ever been, times a hundred! And you just can barely keep your eyes open. If you ever had that experience driving on the highway and you can barely keep your eyes open? It's similar to that.”*

In this way, Jamila was unprepared for the embodied effects of using xylazine. She did not have the opportunity—like other PWUD in her community—to first hear about it through word of mouth, during service provision, in the media, and/or on the internet. Though Jamila did stop by the harm reduction center every day to get some food and sterile supplies, she did not access xylazine education there. She further explained that she had not been seen by a doctor in over a year because she was “too busy ripping and running.” Though Jamila was connected to some services, her experience mirrors that of PWUD who are at the margins (i.e., those who might not be connected to services, technology, etc.). In this way, finding ways by which PWUD at the margins can learn about xylazine before they are exposed to it in their supply is important.

#### Theme 3: Participants sometimes carried misconceptions about xylazine

Provided that participants often learned about xylazine from disparate sources (e.g., from word of mouth, from a TikTok) and through their own experiences using this drug, they occasionally carried some misconceptions about xylazine with them. Table 6 provides short examples of common misconceptions participants held about (a) xylazine’s taste and smell, when xylazine is tasteless and odorless, (b) xylazine’s color, when xylazine is colorless, and (c) the cause(s) of xylazine-associated wounds. For instance, consider Rob, a White man in his forties, who described being able to smell xylazine in his fentanyl supply (see Table 6). When asked if he would ever be interested in getting his drugs checked for xylazine at his local harm reduction center, he stated,

*“I just smell them in advance. Other people come [to the harm reduction center] and check.”*

**Table 6:** Common misconceptions participants held about xylazine

Topic area	Example quotes	
*Xylazine's smell / Taste*		
		"Smells funny. You smell a chemical."—Rob, White Male, 30's
		"The more bitter it is, the stronger it's gonna be."—Mark, White Male, 40's
*Xylazine's color*		
		"When it is blue, it has got that stuff in it."—Jamila, Other Raced Female, 50's
		"For me, it's a powder substance, purplish in color"—Derek, White Male, 40's
*Xylazine-associated wound causes*		
		"Krokodil" - Jorge, Other Raced Male, 40's
		"Brown recluse [, a spider bite]"—Pedro, Other Raced Male, 40's
		"MRSA" - Edward, White Male, 40's

In this way, even seemingly small misconceptions could lead participants like Rob to believe they would not benefit from drug checking services. Though he might be able to smell some other drug or cut that is commonly in the drugs found from the person he buys drugs from, this likely will not hold if Rob were to switch suppliers. Thus, debunking misconceptions is an important part of xylazine-focused education initiatives, as PWUD may have created their own harm reduction measures.

#### Theme 4: Participants expressed interest in many ways of learning more about xylazine

Throughout the interview process, participants reported wanting to learn about many facets of xylazine and in many ways. To help capture this complexity, Table 7 summarizes just some of the many ideas participants expressed when asked about their preferred learning styles and interests. Some participants expressed that *how* this information was portrayed was equally important to *what* information was portrayed. For instance, Whitney, a White woman in her early thirties explained that in regard to educational materials,

*“It would be cool to [include] some stories of people that have lived experience, maybe a little picture of them, and a little blurb about how they got through it and came out on the other side.”*

**Table 7:** Participants' ideas about educational materials on xylazine

Topic area	Example quotes	
*Location*		
		"Methadone clinics" - Christopher, White Male, 30's
		"Doctors' offices" - Whitney, White Female, 30's
		"[Harm reduction center]" - Olivia, White Female, 40's
		"Shelters" - Jorge, Other Raced Male, 40's
		"Bus stops... bodegas... community centers"—Jamila, Other Raced Female, 50's
		"Dispensaries" - Jack, White Male, 40's
		"Library" - Meredith, White Female, 40's
		"Telephone poles" - Isaiah, Black Male, 50's
		"Train stations" - Travon, Black Male, Unknown Age
*Medium*		
		"A documentary" - Jorge, Other Raced Male, 40's
		"A video" - Olivia, White Female, 40's
		"A pamphlet" - Emily, White Female, 30's
		"A magazine" - Whitney, White Female, 30's
		"A poster" - Travon, Black Male, Unknown Age
		"A flyer" - Edward, White Male, 40's
		"Workshops... trainings"—Isaiah, Black Male, 50's
		"A moderated forum" - Nolan, White Male, 30's
		"Classes or sessions with staff"—Hassan, Black Male, 30's
		"A billboard... a helpline" - Alejandro, White and Hispanic Male, 60's
*Content*		
		"How much Narcan they're going to need"—Barbara, White Female, 60's
		"How it takes a toll on your body"—Rodney, Black Male, 50's
		"The cost of xylazine" - Travon, Black Male, Unknown Age
		"What it looks like and what it tastes like"—Meredith, White Female, 40's
		"What is the purpose and what are the effects"—Edward, White Male, 40's
		How it can affect your health"—Sophia, White Female, 40's
		"Is it in weed" - Rob, White Male, 30's

Thus, it was important for Whitney and other participants to not only see themselves (i.e., PWUD) represented in educational materials but to see that it is possible to use xylazine and continue living (e.g., have a wound and have it healed). This type of education, focused on harm reduction and lived experience with non-stigmatizing language at the center, is also important for PWUD.

## Discussion

This study demonstrated that people who are unemployed, engaged in harm reduction services, and/or using fentanyl are more likely to report xylazine use and, congruously, are more likely to have higher knowledge about xylazine and XROs. Data from other CT sources, including community-based drug checking and fatal overdose toxicology, have consistently demonstrated that xylazine is almost exclusively found in combination with fentanyl [16, 18]. Accordingly, it is not surprising that we found that PWUD who use fentanyl were more likely to report xylazine use or exposure. However, our study novelly demonstrates other factors that may be associated with xylazine use or exposure: unemployment and harm reduction service engagement.

### Data integration

Given the cross-sectional nature of this study design, claims about causality or directionality cannot be drawn from quantitative data alone, but the qualitative data suggest potential explanations for some of the significant correlations found. For instance, our data integration suggests that xylazine use may lead to heavy sedation which interferes with one’s ability to go to or be active at work (i.e., leading to or exacerbating existing unemployment). Employment was discussed frequently by PWUD in the interviews, as many indicated that xylazine was different from other drugs they had experience using in the past (e.g., heroin, fentanyl) in such that it was difficult to maintain a sense of control over daily functioning (e.g., driving to work, staying awake on the job) while exposed to xylazine, primarily due to the heavy sedation. Thus, providing specific xylazine education/services to those who are employed and using fentanyl in communities where xylazine is increasingly prevalent may be important. One option may be to provide xylazine test strips to PWUD, so they have an opportunity to know if their fentanyl has xylazine in it during work hours to avoid unknowing exposure. PWUD may also try to limit use of xylazine-positive fentanyl to only the end of the day when one can sleep. It may also be effective for PWUD to hear stories from other PWUD in education initiatives about being unable to continue working when exposed to xylazine in their fentanyl. Regardless, the process of compiling and presenting information in education initiatives can and should actively involve PWUD. This effort must involve local considerations, as what might work best for our participants in the context of our local supply might not work best for other communities.

Additionally, while quantitative data suggest that either harm reduction service engagement is an indicator for drug use patterns that may put individuals at higher risk of xylazine exposure or that xylazine exposure urges people to seek harm reduction services (e.g., seeking wound care), our qualitative data suggests that both correlative directions are occurring and possible. Thus, harm reduction organizations should be prepared to engage people who are newly seeking services due to novel experiences related to xylazine.

Most importantly, this study signals that there are at least two main groups of PWUD with different educational needs that should be targeted for xylazine education:

### Target education group 1: Those using fentanyl

The first group of PWUD are those using fentanyl; these folks are likely to already be engaged in harm reduction services and have higher awareness and knowledge about xylazine’s impact through either lived/living experience, word of mouth in their communities, or education via harm reduction organizations. Information provided to this group should expand beyond basic awareness and be aimed at (a) decreasing existing knowledge gaps, (b) increasing confidence in managing xylazine in their drug supply and XROs and (c) providing less stigmatizing access to services/materials to treat such XROs. As such, education initiatives may focus on topics such as drug checking, managing prolonged sedation, and wound care.

### Target education group 2: Those not using fentanyl

The second group is people not using fentanyl or those who are not aware that they might be using fentanyl-adulterated substances. Their specific educational needs require building initial awareness of the chance of accidental xylazine exposure and basic knowledge about XROs. Importantly, we found that only half of those who had no reported xylazine use were aware of xylazine prior to the study; thus, it is unclear whether these individuals had actually never been exposed to xylazine or if they just lacked awareness to conclude that they had been exposed. Regardless, and unsurprisingly, those who reported no xylazine use knew very little about xylazine and XROs. For instance, more than 90% of this group did not know that xylazine decreased one’s heart rate. Further, this group reported lower engagement in harm reduction services. Thus, education initiatives aimed at increasing awareness among this second group should include PWUD who are not engaged in harm reduction services; this might be an opportunity for healthcare providers who see PWUD outside of the context of their drug use to introduce xylazine and XROs, and to recognize and compensate PWUD for the work they do in information sharing within their communities.

### Goals for educational initiatives

One of the primary takeaways from this work was that PWUD sought out and learned about xylazine through numerous mechanisms, and thus accurate content can and should be readily available for PWUD to consume through multiple mechanisms, (1) in the places they wish to consume it, (2) in mediums that match the given place and time, and (3) with content that is driven by the target education group’s needs. This might look like, for instance, a harm reduction organization setting aside time to bring in a trainer during a group meeting to talking about drug checking and XROs. It may also look like hanging flyers about xylazine in a methadone clinic and using the flyer as a way to start conversations with patients who are using fentanyl.

Further, PWUD wanted to be engaged in learning and building knowledge. Educational materials should be informed by the personal experiences of PWUD to help other PWUD contextualize what one might expect from using xylazine. Our participants made it clear that this material (i.e., personal stories) is something they do in fact seek out when trying to build a knowledge base around xylazine and confidence around managing it in their supply; it would be best to curate this educational material to ensure it is free, accessible, community-informed, and as accurate and up to date as possible.

We assert that in order to create effective education initiatives, both clinical and harm reduction service providers must create stigma-free and trusting environments where PWUD are comfortable to talk about what they are experiencing. Our participants expressed wanting to speak truthfully about the hard realities of xylazine exposure (i.e., XROs) and be able engage in conversations with trusted partners. However, this was not expressed without certain hesitancies around clinical environments, where some participants feared they may be judged or shamed for their use. Certainly, there is well-documented evidence that PWUD can face difficulties seeking healthcare—especially care related to their drug use—as physical markings (e.g., abscesses, sores, etc.) can lead to feelings of shame and stigma [19, 20, 21, 22]. The severity of xylazine-associated wounds is likely to intensify such stigma [10, 23, 24]. If clinical settings aim to have successful education initiatives around xylazine, they must first aim to ensure they working to have safe, comfortable, and stigma-free spaces.

Alternatively, some PWUD may seek such truths from public health messaging through news channels or social media that local clinical and harm reduction service providers do not have control over. Such mediums reach PWUD in important ways that can inform harm reduction best practices; thus, it is important that these mechanisms get the messaging right and are sensitive to their diverse audiences. Many of our participants took comfort in seeing others like them on their screens, but misrepresentation and misinformation can be harmful. News outlets have the responsibility to recognize that PWUD are also consumers of their content, and thus should make every effort to report accurately, use non-stigmatizing language (e.g., avoid use of terms like “zombie” when describing xylazine-associated sedation), and connect PWUD to evidence-based resources whenever possible [25, 26].

Regardless of target education group, we have also demonstrated that misconceptions about xylazine among PWUD needs to be addressed in education initiatives to ensure people have accurate information from trusted sources. Yet, it is also important to create time for trainers to acknowledge and explore current uncertainties related to xylazine and XROs (e.g. what exactly causes xylazine associated wounds?). While PWUD may hold misconceptions from disparate sources or lived experiences, they also—uniquely—likely hold the answers to many of the key questions in the field of harm reduction and addiction medicine. This asymmetric knowledge can be transformed into power, if PWUD are given appropriate time, resources, trust, and space to do so. Differentiating what is a misconception among PWUD (e.g., xylazine has a taste or color) and what is a novel finding (e.g., xylazine may lead to wounds) may be difficult, but such is the work of harm reduction and clinical research.

### Future research

The quantitative arm of this study, and our previous mixed-methods study on CT healthcare provider perspectives revealed gaps in existing knowledge about xylazine [11]; and the qualitative arms of each study indicated that people are asking questions for which we currently do not have the answers. Thus, both providers and PWUD will benefit from active efforts by researchers to fill gaps in education and practice. These areas include, but are not limited to, improving understanding of xylazine withdrawal management, the causative factors and prevention strategies for wounds, and strategies to increase safety during sedation. Future research is crucial to assess the impact of implementing enhanced educational training for PWUD, service providers, and harm reductionists.

### Limitations

While this study had numerous strengths, some key limitations should be noted. Primarily, we are analyzing reported use of xylazine, not objectively verified use of xylazine; accordingly, it is important to take this into account when interpreting results. Though qualitative data suggest that many people from the reported use group were aware of their status based on the particular sensation of prolonged xylazine-induced sedation, engagement in drug checking, conversations with people who they were buying from, and urine screens during substance use treatment; there is likely some misclassification of self-reported xylazine use (e.g., PWUD in the ‘no reported use’ group may actually have used xylazine previously but unknowingly).

Secondly, there is potential for selection bias away from the null (i.e., the groups of xylazine exposed and xylazine not exposed know the same amount), created via the use of convenience sampling from visitors to service providers. CT is home to many dedicated harm reduction organizations that serve PWUD, providing life-saving supplies, building community, sharing educational materials, and connecting people to other needed services (i.e., housing, healthcare, transportation, legal assistance). The fact that almost three-fourths of our sample were aware of xylazine, which only emerged in CT in 2019, is a testament to the work of the harm reduction organizations with whom we collaborate and the support PWUD provide each other to increase safety [12, 13]. In fact, our study found higher levels of xylazine awareness and knowledge among PWUD than previous studies conducted in other states [27, 28, 29]. Overall, this means that other communities without such access to harm reduction services (and previous exposure to/awareness of xylazine), there may be smaller differences in xylazine knowledge and confidence between those with and without reported lifetime xylazine exposure. Thus, these findings may limit generalizability and/or transferability to other communities of PWUD outside of CT or even the Northeast; in fact, our sample may not even be representative of all PWUD in CT. For instance, factors related to recruitment likelihood could be correlated with the primary outcome (i.e., engagement in harm reduction services and xylazine use).

Lastly, our small sample size might mean that our study had insufficient power to detect differences between subgroups should they exist. Additionally, small cell sizes in certain measurements makes it more difficult to ascertain point estimates with high confidence.

## Conclusions

Our mixed methods study is the first, to our knowledge, to explore what knowledge and confidence gaps among PWUD need to be filled via xylazine-focused education initiatives, and whom such initiatives should focus on. Harm reduction organizations, PWUD themselves, and healthcare providers can serve as conduits for information provision to PWUD; people with varying exposure to xylazine will have different levels of need for education. However, xylazine-specific education and training should be free, accessible, harm-reduction oriented, stigma-free, and include the voices of PWUD.

## Supplementary Information

Additional file1 (PNG 910 KB)

## Data Availability

The datasets generated and/or analyzed during the current study are not publicly available to protect participant privacy and confidentiality.

## Abbreviations

CT: Connecticut
PWUD: People who use drugs
XRO: Xylazine related outcomes
