# Supplementary figures and images for: Zeroing in on xylazine - a mixed methods study to explore correlates of reported xylazine use and educational gaps among people who use drugs
Source: Harm Reduct J. 2026 Apr 3;23:88. doi: 10.1186/s12954-026-01437-7 (PMC13173959; doi:10.1186/s12954-026-01437-7)

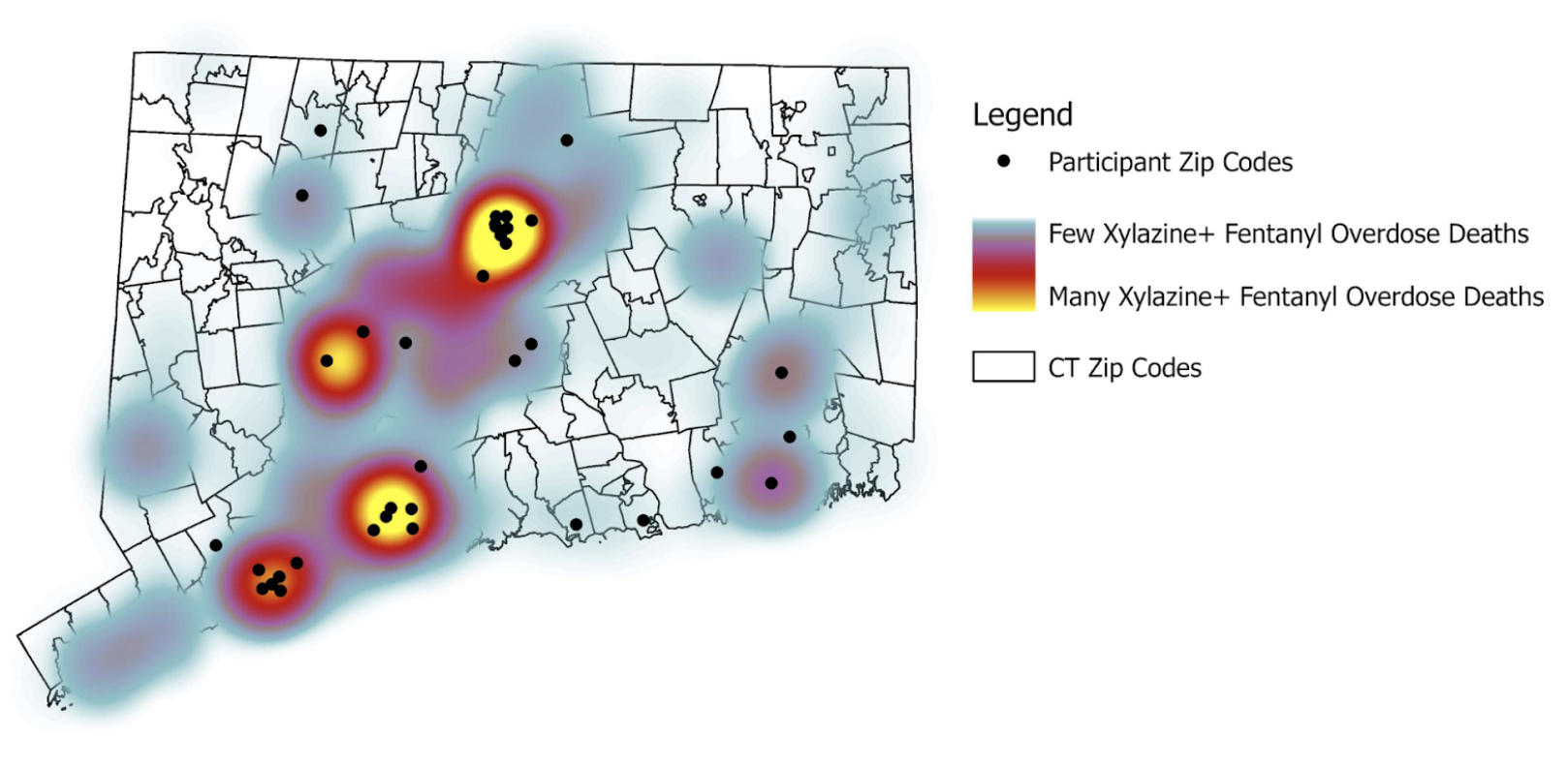

Supplement: Supplementary file 1 — Additional file1 (PNG 910 KB) [file 12954_2026_1437_MOESM1_ESM.png]
